# Germline mutation rates in young adults predict longevity and reproductive lifespan

**DOI:** 10.1038/s41598-020-66867-0

**Published:** 2020-06-19

**Authors:** Richard M. Cawthon, Huong D. Meeks, Thomas A. Sasani, Ken R. Smith, Richard A. Kerber, Elizabeth O’Brien, Lisa Baird, Melissa M. Dixon, Andreas P. Peiffer, Mark F. Leppert, Aaron R. Quinlan, Lynn B. Jorde

**Affiliations:** 1 Department of Human Genetics, University of Utah, Salt Lake City, UT United States; 2 Population Science, Huntsman Cancer Institute, University of Utah Health, Salt Lake City, UT United States; 3 Department of Health Management & Systems Sciences, University of Louisville, Louisville, KY United States; 4 Department of Pediatrics, University of Utah, Salt Lake City, UT United States; 5 Department of Biomedical Informatics, University of Utah, Salt Lake City, UT United States; 6 USTAR Center for Genetic Discovery, University of Utah, Salt Lake City, UT United States

**Keywords:** Comparative genomics, Mechanisms of disease, Genomic instability, DNA damage and repair, Predictive markers, Infertility

## Abstract

Ageing may be due to mutation accumulation across the lifespan, leading to tissue dysfunction, disease, and death. We tested whether germline autosomal mutation rates in young adults predict their remaining survival, and, for women, their reproductive lifespans. Age-adjusted mutation rates (AAMRs) in 61 women and 61 men from the Utah CEPH (Centre d’Etude du Polymorphisme Humain) families were determined. Age at death, cause of death, all-site cancer incidence, and reproductive histories were provided by the Utah Population Database, Utah Cancer Registry, and Utah Genetic Reference Project. Higher AAMRs were significantly associated with higher all-cause mortality in both sexes combined. Subjects in the top quartile of AAMRs experienced more than twice the mortality of bottom quartile subjects (hazard ratio [HR], 2.07; 95% confidence interval [CI], 1.21–3.56; p** =** 0.008; median survival difference** =** 4.7 years). Fertility analyses were restricted to women whose age at last birth (ALB) was** ≥** 30 years, the age when fertility begins to decline. Women with higher AAMRs had significantly fewer live births and a younger ALB. Adult germline mutation accumulation rates are established in adolescence, and later menarche in women is associated with delayed mutation accumulation. We conclude that germline mutation rates in healthy young adults may provide a measure of both reproductive and systemic ageing. Puberty may induce the establishment of adult mutation accumulation rates, just when DNA repair systems begin their lifelong decline.

## Introduction

The somatic mutation theory of ageing^1^ proposes that somatic mutations accumulate throughout life, resulting in apoptosis, cellular senescence, tumorigenesis, or other cellular pathologies, followed by tissue dysfunction, chronic disease, and death. Several monogenic progeroid syndromes are characterized by DNA repair deficiencies, increased somatic mutation rates, early onset of ageing-related phenotypes, and shortened lifespans^2^, strongly supporting the somatic mutation theory of ageing. In healthy individuals DNA damage is continuous^3^, and while most of it is repaired, several classes of DNA damage are known to accumulate through adulthood in both sexes^4–7^, though at higher rates in men^8^. Recent studies^9–11^ have reported that somatic mutations in blood nuclear DNA can be detected and quantified in nearly all healthy middle-aged and older individuals, and that higher somatic mutation levels predict higher all-cause mortality, providing further support for the somatic mutation theory of ageing.

As yet, it is unknown whether mutation accumulation in germline and/or somatic tissues during normal ageing is an important limiter of reproductive lifespan in women. Fertility in healthy women declines after age 30^12–14^, ending with menopause between ages 40 and 60. Several studies suggest that an individual’s reproductive ageing is correlated with their systemic ageing. Selection for late fecundity in females in Drosophila melanogaster over many generations resulted in increased longevity in both sexes^15^. Women whose age at last birth (ALB) is over 40 years live significantly longer than women whose ALB is younger^16^; and brothers and sisters of women with an older ALB tend to be long-lived^17^. Women who deliver higher numbers of live births have longer lifespans^18–21^. Finally, older ages at natural menopause are associated with longer lifespans^22^.

Important questions remain regarding mutation accumulation in normal ageing populations: 1) How early in life do levels of mutation accumulation predict remaining longevity? 2) Do levels of mutation accumulation early in life predict reproductive lifespans? And 3) do somatic and germline mutation accumulation rates rise after puberty, as predicted by the evolutionary biology principle that the force of natural selection to maintain robust health should begin to decline once the reproductive phase of life is attained^23,24^?

It is reasonable to hypothesize that mutation accumulation rates in healthy individuals increase after puberty, since 1) blood levels of insulin-like growth factor I (IGF-I) peak at puberty^25^, suppressing the FOXO transcription factors and the DNA repair genes that FOXO upregulates^26^, and 2) DNA repair systems are known to decline throughout adult life^27^. Developmental deficiency of the GH/IGF-1 axis in dwarf mice keeps IGF-1 levels in blood low, prevents the normal decline in DNA repair of adulthood, and significantly extends lifespan^28^. Also, polygenic risk scores for later onset of puberty in humans are associated with longer lifespans in both sexes^29^; and later puberty is associated with decreased all-cause mortality in women^30,31^, reduced risk of cancer in both sexes^32^, and later menopause^33–36^. All of these associations are expected if mutation accumulation rates rise after puberty and contribute to both systemic and reproductive ageing.

While mutation accumulation rates are much lower in germline than in soma^37^, many of the effectors of DNA damage and the repair systems defending against it are shared across tissues^1,3,27,38,39^. Therefore, ranking sex- and age-matched individuals by their germline mutation accumulation rates may effectively also rank them by their somatic mutation accumulation rates. Recently, we analyzed blood-DNA-derived whole genome sequencing (WGS) data from 41 three-generation Utah CEPH families^40–42^ (Supplementary Fig. 1); in 61 Generation II individuals we identified *de novo mutations* (DNMs) whose origins could be attributed specifically to the male and female germlines of their parents (61 Generation I couples). Here we derive from these mutation counts parental-age-adjusted germline autosomal mutation rates for these 122 Generation I subjects and test whether the mutation rates are associated with two clinically important life history traits in those same Generation I individuals: lifespan in both sexes and the duration of childbearing in women, as would be expected if germline mutation accumulation reflects the rate of both systemic and reproductive ageing. We also investigate the hypothesis that puberty initiates the establishment of adult germline mutation accumulation rates following a prepubertal quiescent period when mutation burdens may be plateaued.

## Results

### Survival analyses

**Figure 1:**
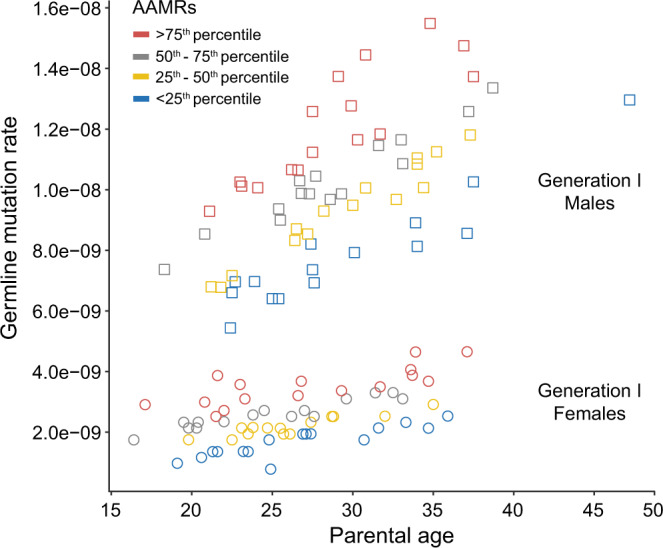
The frequency or rate of mutations in the germ cells of young adults increases with age and can vary more than 2-fold between sex- and age- matched individuals. Germline mutation rates were measured as (#germline autosomal mutations)/(#diploid autosomal callable base pairs). The single data point plotted for each of the 61 Generation I males (squares) and 61 Generation I females (circles) is derived from *de novo* mutations discovered in a single one of their offspring. After adjusting for the effects of parental age, the mutation rate of each individual was assigned to a quartile of Age-Adjusted Mutation Rate (AAMR), with each quartile indicated by one of the four colors in the graph. Differences between Generation I individuals in their germline mutation rates are unlikely to be due to differences in the presence or absence or degree of progression of various terminal illnesses, since all Generation I subjects survived more than 20 years past the age at which they transmitted these germ cell mutations to their offspring. Furthermore, it is unlikely that any of the mutations analyzed here are strongly deleterious, since all Generation II individuals in whom the *de novo* mutations were identified are known to have reached maturity and had several children of their own. (Adapted from Sasani *et al*.^40^, Fig. 2a).

The demographic characteristics of the 122 Generation I subjects whose germline mutations have been counted in the genomes of their offspring (61 Generation II individuals) are presented in Supplementary Table 1. Generation I germline mutation rates, (#germline autosomal mutations)/(#diploid autosomal callable base pairs), derived from DNMs discovered in a single offspring of each Generation I individual, and the ages of the Generation I individuals when those children were born, are plotted in Fig. 1. These cross-sectional data show that germline mutation burdens in this cohort increase with parental age in both sexes, as has been previously reported^40^. We first aimed to test, among sex-matched individuals of similar parental age, whether those with more germline mutations tend to be shorter-lived than those with fewer mutations. Therefore, we regressed germline autosomal mutation rates on parental age using a generalized linear model and used the resulting residuals to represent age-adjusted mutation rates (AAMRs). AAMRs as either a continuous variable or as a categorical variable were then tested for their association with overall survival and cause-specific mortality. All categorical comparisons of survival used <25^th^ percentile of AAMRs as the reference category.

Each data point in Fig. 1 is color-coded to denote the quartile of AAMRs to which it was assigned. The median parental age of the subjects in each quartile of AAMRs is given in Supplementary Table 2. Associations of AAMRs with all-cause mortality, cardiovascular disease (CVD) mortality, and non-CVD mortality were analyzed in both sexes combined and in each sex separately (Table 1). CVD mortality includes deaths from heart disease, stroke, and hypertension. This approach revealed differences between men and women in the causes of mortality most strongly associated with increasing germline mutation rates.

**Table 1:** Associations of germline mutation rates with mortality in 122 Generation I individuals.

	Age-adjusted germline mutation rates	All-cause mortality		CVD mortality		Non-CVD mortality	
		HR (95% CI)	p	HR (95% CI)	p	HR (95% CI)	p
Both sexes	Continuous	1.28 (1.05, 1.56)	***0.015***	1.14 (0.87, 1.49)	0.355	1.49 (1.12, 2.00)	***0.007***
	25^th^-50^th^ percentile	1.51 (0.88, 2.59)	0.136	1.67 (0.79, 3.50)	0.177	1.41 (0.63, 3.14)	0.406
	>50^th^-75^th^ percentile	1.59 (0.94, 2.70)	0.087	1.29 (0.57, 2.91)	0.545	2.07 (1.01, 4.24)	***0.047***
	> 75^th^ percentile	2.07 (1.21, 3.56)	***0.008***	1.28 (0.56, 2.93)	0.559	3.24 (1.57, 6.68)	***0.001***
	Trend test	1.25 (1.06, 1.48)	***0.009***	1.05 (0.82, 1.34)	0.694	1.48 (1.18, 1.87)	***0.001***
Males	Continuous	1.40 (1.04, 1.87)	***0.025***	1.39 (0.94, 2.07)	0.101	1.45 (0.94, 2.22)	0.092
	25^th^-50^th^ percentile	1.24 (0.58, 2.65)	0.578	1.62 (0.55, 4.76)	0.383	1.11 (0.37, 3.37)	0.849
	>50^th^-75^th^ percentile	1.80 (0.85, 3.83)	0.125	2.64 (0.86, 8.17)	0.091	1.45 (0.51, 4.14)	0.483
	> 75^th^ percentile	2.21 (1.03, 4.76)	***0.043***	2.39 (0.77, 7.46)	0.132	2.27 (0.79, 6.51)	0.126
	Trend test	1.32 (1.03, 1.68)	***0.026***	1.35 (0.96, 1.90)	0.084	1.31 (0.93, 1.85)	0.122
Females	Continuous	1.22 (0.93, 1.59)	0.158	0.74 (0.49, 1.12)	0.152	1.76 (1.24, 2.51)	***0.002***
	25^th^-50^th^ percentile	2.04 (0.91, 4.59)	0.085	1.63 (0.56, 4.71)	0.368	2.17 (0.61, 7.64)	0.230
	>50^th^-75^th^ percentile	1.44 (0.67, 3.10)	0.350	0.43 (0.11, 1.69)	0.225	2.91 (1.06, 8.03)	***0.039***
	> 75^th^ percentile	1.97 (0.88, 4.38)	0.097	0.41 (0.10, 1.60)	0.199	5.16 (1.79, 14.93)	***0.002***
	Trend test	1.18 (0.93, 1.50)	0.180	0.70 (0.47, 1.04)	0.081	1.68 (1.21, 2.33)	***0.002***

Associations with mortality of age-adjusted mutation rates (AAMRs) treated as a continuous or categorical variable were analyzed in both sexes combined and in each sex separately. In each section (Both sexes, Males, and Females) the first row (Continuous) presents the effects on the Hazard Ratio (HR) of a one standard deviation increase in AAMRs. The second, third, and fourth rows present the mortality risks for subjects with increasing quartiles of AAMRs, expressed relative to the mortality risks for the lowest quartile (<25^th^ percentile). The thresholds for the AAMRs are Males: 25% = −1.2385992, 50% = −0.1078028, 75% = 1.2402645; Females: 25% = −0.61610326, 50% = −0.09220471, 75% = 0.41736567. These thresholds were calculated using all 122 Generation I subjects. CI = Confidence Interval.

After adjusting for birth year and sex in Cox proportional hazard regression models, the analysis of both sexes combined revealed that a one standard deviation increase in AAMRs was associated with higher all-cause mortality (hazard ratio [HR], 1.28; 95% confidence interval [CI], 1.05–1.56; p = 0.015), and higher non-CVD mortality (HR, 1.49; 95% CI, 1.12–2.00; p = 0.007), but not associated with CVD mortality (Table 1). In the categorical analyses of both sexes combined, subjects in the top quartile of AAMRs experienced more than twice the all-cause mortality of bottom quartile subjects (hazard ratio [HR], 2.07; 95% confidence interval [CI], 1.21–3.56; p = 0.008), and more than three times the non-CVD mortality (HR, 3.24; 95% CI, 1.57–6.68; p = 0.001). In tests for trend in both sexes combined, the associations of increasing quartiles of AAMRs with increasing all-cause and non-CVD mortality were both statistically significant. (When germline mutation rates were adjusted for parental age by simply dividing each subject’s mutation rate by their parental age, similar, though less robust associations with all-cause mortality risks were observed: Supplementary Table 3).

In men a one standard deviation increase in AAMRs was significantly associated with higher all-cause mortality (HR, 1.40; 95% CI, 1.04–1.87; p = 0.025), but not associated with either non-CVD or CVD mortality (Table 1). Men in the top quartile of AAMRs experienced more than twice the all-cause mortality of men in the bottom quartile (hazard ratio [HR], 2.21; 95% confidence interval [CI], 1.03–4.76; p = 0.043). Tests for trend in men showed a significant association of increasing quartiles of AAMRs with increasing all-cause mortality, but not with CVD mortality or non-CVD mortality. These categorical analyses suggest that in men, both CVD mortality and non-CVD mortality contribute to the significant association of AAMRs with all-cause mortality.

In women a one standard deviation increase in AAMRs was significantly associated with higher non-CVD mortality (HR, 1.76; 95% CI, 1.24–2.51; p = 0.002), but not associated with either all-cause mortality or CVD mortality (Table 1). Women in the top quartile of AAMRs experienced more than five times the non-CVD mortality of women in the bottom quartile (HR, 5.16; 95% CI, 1.79–14.93; p = 0.002). Tests for trend in women showed a significant association of increasing quartiles of AAMRs with increasing non-CVD mortality, but not with all-cause mortality or CVD mortality. These categorical analyses suggest that in women, the association of AAMRs with mortality appears to be driven almost entirely by their association with non-CVD mortality.

The higher germline mutation counts and higher rates of accumulation of germline mutations in men vs. women have been attributed to the male, but not the female, germline undergoing rounds of genome copying and cell division throughout adulthood that generate replication errors; in addition there appear to be higher rates of unrepaired DNA damage (independent of genome replication) in male vs. female germlines^43^. How these sex differences in germline mutation dynamics may relate to the different patterns of associated cause-specific mortality in men vs. women remains to be elucidated.

**Figure 2:**
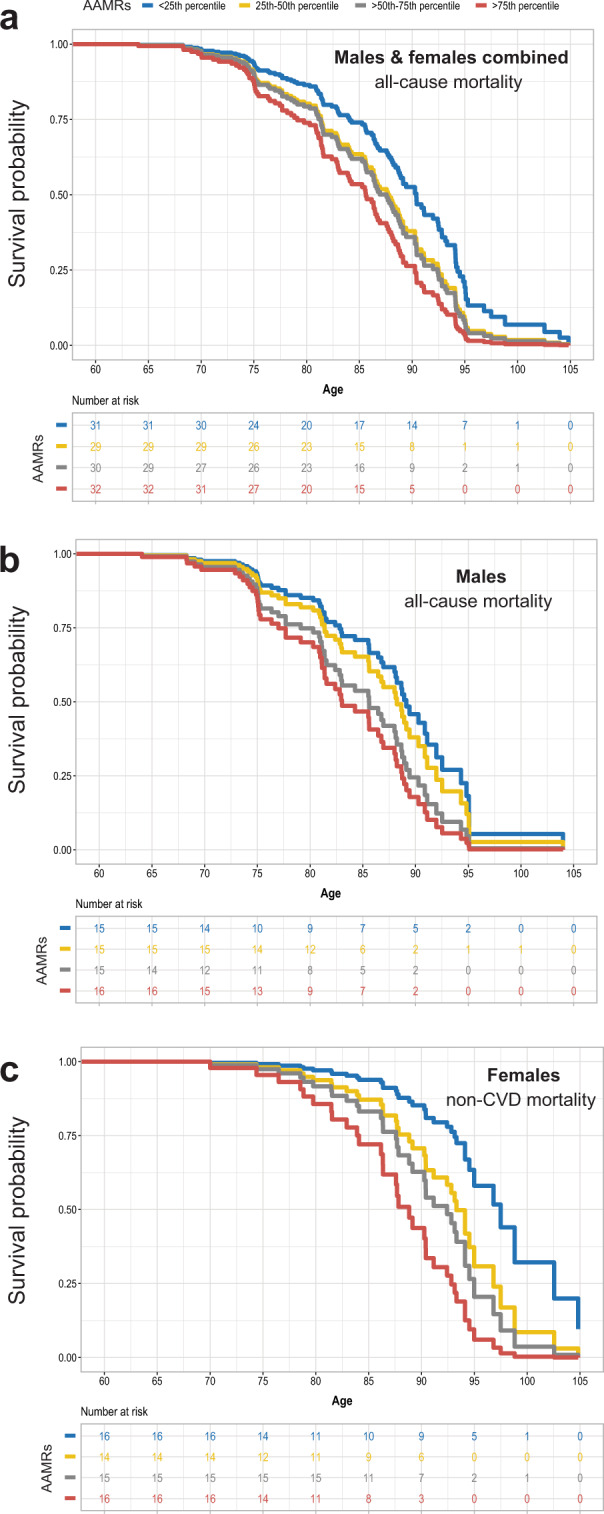
Predicted survival curves by quartiles of age-adjusted germline mutation rates. Parental age and birth year were fixed to their median values (25 years and 1912, respectively) based on the fitted model in Table 1. (**a**) both sexes combined, all-cause mortality; (**b**) males only, all-cause mortality; (**c**) females only, non-cardiovascular disease (non-CVD) mortality. AAMRs: age-adjusted mutation rates, with quartiles color-coded as in Fig. 1.

The adjusted survival curves in Fig. 2 are color-coded by quartile of AAMRs to match the scheme used in Fig. 1. The median survival advantage for all-cause mortality in the analysis of both sexes combined, for those with germline mutation rates <25th percentile vs. >75th percentile, was approximately 4.7 years. For male all-cause mortality, the median survival advantage for bottom quartile vs. top quartile was approximately 6 years. For female non-CVD mortality, the median survival advantage for bottom vs. top quartile was approximately 8 years. These associations with survival, similar in magnitude to the effects on survival of smoking or physical activity^44^, are not unexpected if germline mutation rates indeed reflect rates of ageing generally and rates of ageing in healthy individuals vary more than 3-fold^45,46^. We hypothesize that individuals with higher germline AAMRs also accumulate somatic mutations at higher rates systemically, resulting in an earlier onset of multiple ageing-related lethal diseases, consistent with these survival data.

### Cancer incidence

While somatic mutations are known contributors to tumorigenesis^47^, a connection between mutation accumulation rates (in either somatic or germline tissues) in healthy young adults and cancer risks has not yet been established. In the set of 122 Generation I individuals, there were 16 women and 18 men who received at least one cancer diagnosis in their lifetimes, though cancer was the cause of death for only 8 individuals. We sought to test the hypothesis that in the full cohort of 122 subjects, lower AAMRs would be associated with lower age-specific cancer risks (Supplementary Table 4). Associations were tested with AAMRs treated as a continuous variable and also as a categorical variable where cancer risks in the higher tertiles of mutation rates are compared to cancer risk in the lowest tertile. Tertiles rather than quartiles were analyzed to provide more stable risk estimates, given the small number of cancer cases. No significant associations of germline mutation rates with cancer risk were found.

### Fertility of women

We hypothesized that germline mutation accumulation may contribute to oocyte atresia, lower rates of fertilization, higher rates of miscarriage, and/or earlier menopause, and consequently, shorter reproductive lifespans. Since cessation of childbearing prior to age 30 is unlikely due to reproductive ageing^12–14^, these analyses were restricted to the 53 Generation I women with age at last birth ≥ 30. We tested whether women with higher rather than lower AAMRs gave birth to fewer children, and had a younger ALB (Table 2). Among these women, those in the top two thirds of AAMRs had fewer live births than those in the bottom third (p = 0.018), and higher mutation rates were significantly associated (p = 0.036) with a younger ALB ( < 25th percentile, 34.8 years).

**Table 2:** Associations of germline mutation rates with reproductive lifespan in 53 Generation I women with ALB ≥ 30 years.

Age-adjusted germline mutation rates	Number of live births				Age at last birth < 25^th^ percentile	
	Est	SE	Z	p	RR (95% CI)	p
Continuous	−0.12	0.08	−1.63	0.104	2.12 (1.05, 4.26)	**0.036**
≥33^rd^ percentile	−0.27	0.11	−2.36	**0.018**	4.27 (0.81, 22.41)	0.086

Associations of AAMRs as a continuous or categorical variable with the number of live births and ALB for the 53 Generation I women with ALB ≥ 30 years. For the categorical analyses, women in the top two thirds of AAMRs were compared to women in the bottom third. Poisson regression was used to assess the association of AAMRs with the number of live births. Logistic regression models were used to assess the association of AAMRs with ALB. These associations were additionally adjusted for birth year of the Generation I woman, and whether she had any live births with missing birth dates in UPDB. The number of live births decreased by 8.73% for each standard deviation increase in the AAMRs (p = 0.104), and women in the top two thirds for AAMRs had significantly fewer live births than those in the bottom third for AAMRs (p = 0.018). The risk of the ALB being below the 25^th^ percentile increased 2.12 times for every standard deviation increase in the AAMRs (95% CI 1.05–4.26), p = 0.036). The 33^rd^ percentile cut point for AAMRs was −0.46200203. The 25^th^ percentile cut point for age at last birth was 34.8 years.

### When are the germline mutation accumulation rates of adulthood established?

Our data suggest that germline mutation accumulation rates in young adults may be a measure of the rate of ageing. Sasani *et al*.^40^ demonstrated (in their Fig. 3c,d) a 3+ fold range of germline mutation accumulation rates in 40 CEPH Generation II parental couples (primarily reflecting the levels and rates of accumulation of mutations in the fathers’ germlines). Taken together these data suggest that the rate of ageing may vary 3-fold between young adults. Recent studies of inter-individual variation in the pace of ageing in young adults, based on measures of physical performance, physiological functioning, cellular and biochemical markers, and periodontal health also found approximately 3-fold variation between individuals^45,46^.

**Figure 3:**
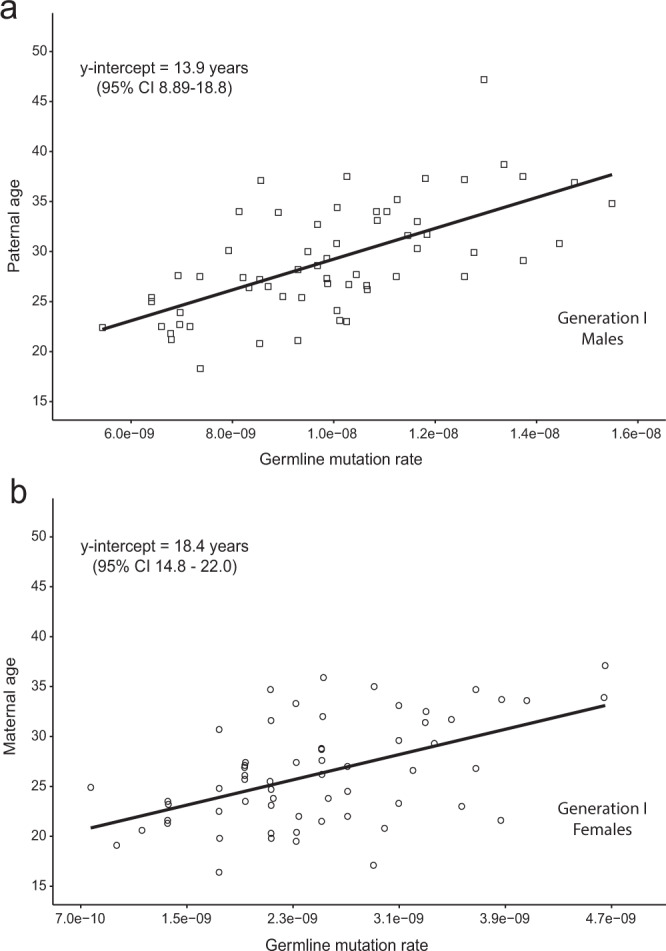
Estimating the age when germline mutation accumulation rates are established. The germline mutation rates plotted in Fig. 1 are again plotted here, but with the x and y axes flipped. Analyzed in this way, the y intercepts of the linear regression lines, when mutation counts would be zero, provide approximate lower bounds for the ages when the observed mutation accumulation rates (slopes of the regression lines) were established: about 14 years for males (panel a) and 18 years for females (panel b). Germline mutation rate = (#germline autosomal mutations)/(#diploid autosomal callable base pairs).

When are the germline mutation accumulation rates of adulthood established? The age at which mutation accumulation begins (i.e. the age when mutation burdens are zero) can be estimated as the x-intercept of a linear regression line fitted to the data in Fig. 1. By this analysis, mutation burden goes to zero about 8.1 years before conception for males, and 2.7 years before conception for females, both of which are biologically impossible scenarios. To explain this, one need only invoke a model whereby mutation rates are higher sometime between conception and adolescence than later in life. Indeed, it was recently reported that somatic mutation rates in human fetal tissues are several-fold higher than in adult tissues of the same types^48^, likely due to the rapid cell growth and proliferation requirements of early development. The dynamics of how the high mutation rates of early development decline across childhood, eventually arriving at the lower rates typical of young adults, is not yet well-characterized. As has been previously suggested by others^49^, we hypothesize that germline mutation burdens may be plateaued during the prepubertal late childhood years, and that adult germline mutation accumulation rates are established around the time of, and perhaps triggered by, puberty.

To investigate this hypothesis, we plotted germline mutation rates (x axis) vs. parental age (y axis) for our Generation I men (Fig. 3a) and women (Fig. 3b). In these analyses the y-intercepts are the ages when the mutation rates would equal zero. We interpret the y intercepts as approximate lower bounds for the ages when the observed mutation accumulation rates (slopes of the regression lines) began: ~18 years for females and ~14 years for males. This timing suggests that puberty may indeed have a causal role in establishing the mutation accumulation rates of adulthood.

**Figure 4:**
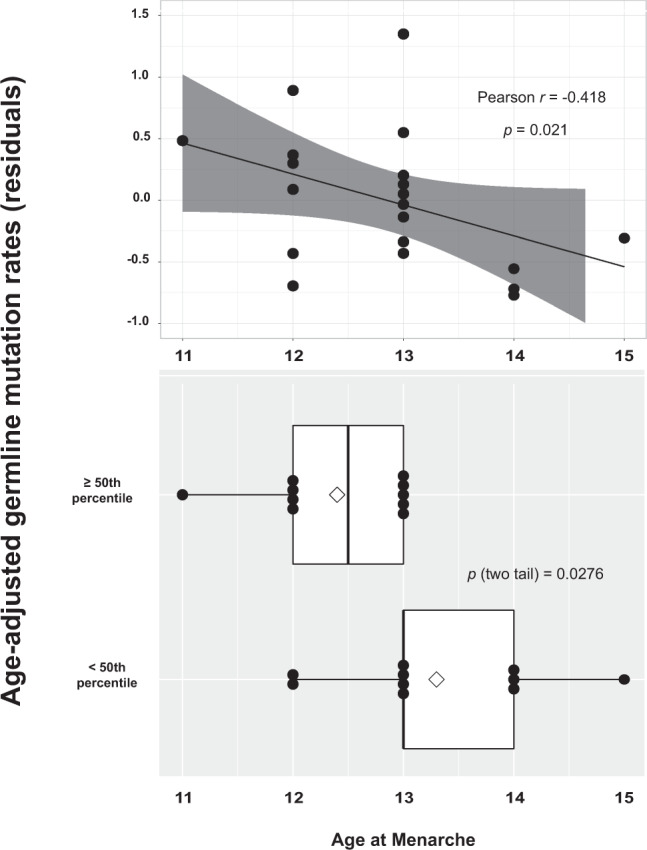
Effects of age at menarche on germline mutation rates in 20 Generation I women. Top panel: linear regression of age at menarche vs. AAMR showing that older ages at menarche are associated with lower AAMRs, Pearson correlation coefficient r = −0.418, p value = 0.021. Bottom panel: box plot of age at menarche by two categories of AAMRs, <50th percentile (<0.0095) and ≥50th percentile (≥0.0095), showing that the mean age at menarche for women in the bottom half for AAMRs (13.3 years) was significantly higher than the mean age at menarche for women in the top half for AAMRs (12.4 years), by a two-tail t test (p  =  0.0276). Diamonds mark the mean ages of menarche.

We then tested another prediction of the hypothesis, that later onset of puberty should be accompanied by delays in mutation accumulation and, therefore, lower AAMRs. Age at menarche data were available for 20 of the Generation I women. We found that older ages at menarche were associated with lower AAMRs (Fig. 4, top panel), Pearson correlation coefficient *r* = −0.418, *p* = 0.021. Also, women below the median AAMR had a higher mean age at menarche than those above the median AAMR (two-tailed t-test, p = 0.028, Fig. 4, bottom panel). Additional studies of age at menarche vs. AAMRs in larger cohorts are needed to test the reproducibility of these weak but statistically significant associations found in our small sample.

Finally, since the range of age of onset of puberty is approximately five years^50^, the hypothesis further predicts that adults with equivalent mutation accumulation rates should vary roughly five years in the age at which they acquire the same number of mutations. We aimed to test this prediction for men using our longitudinal data on germline mutation accumulation in Generation II parental couples, given that the germline mutation burdens and rates of accumulation in couples mainly reflect those of the men, and found the prediction was supported (Supplementary Fig. 2).

Taken together, these results from both sexes suggest a model whereby puberty induces germline mutation accumulation after a relatively quiescent prepubertal period when mutation burdens may be plateaued. The risk of dying is also plateaued and at its lowest level during the prepubertal years^51^. Together, these observations support the hypothesis that ageing begins at or soon after puberty, due to a decline in the force of natural selection to maintain robust health once the reproductive phase of life is attained^23,24^.

## Discussion

Here we have shown that lower sex- and parental-age-adjusted germline mutation rates in young adults are associated with lower all-cause mortality for both sexes, and more liveborn children and older age at last birth for the women. Therefore, germline mutation accumulation rates in young adults may provide a measure, at least in part, of the rates of both reproductive and systemic ageing. To our knowledge this is the youngest age range yet in which a molecular biomarker measured in healthy individuals has been found to predict remaining life expectancy. The strong association of germline mutation rates in young adults with all-cause mortality decades later in this small cohort of research subjects suggests that germline and somatic mutation rates are likely correlated with one another, and that the somatic mutation theory of ageing is correct. We encourage replications of our analyses in additional larger cohorts, especially those with counts of germline mutations and clinical follow-up data already in hand, to determine whether our findings and conclusions will be corroborated.

Though we expected to find significant associations of higher AAMRs with higher cancer risk, we found none. However, the numbers of subjects (n = 122) and cancer diagnoses (n = 34) in our study were small, and the requirement that all 122 subjects had to achieve grandparent status may have selected somewhat for lower cancer risks. Therefore we hope to further investigate this hypothesis in larger cohorts of healthy young adults prospectively followed until first cancer diagnosis.

We have also presented here new analyses of our previously published cross-sectional (Generation I) and longitudinal (Generation II) data on germline mutation accumulation rates in young adults, aimed at estimating the age when adult germline mutation accumulation rates are established. The results from these analyses, together with our novel finding that later menarche is associated with lower germline AAMRs, support a model (Supplementary Fig. 3) whereby germline mutation burdens may be plateaued in the prepubertal years, with the establishment of adult germline mutation accumulation rates occurring sometime during adolescence, perhaps triggered by the onset of puberty. This model is consistent with previous reports suggesting that later puberty is associated with longer lifespans^29–31^, reduced cancer risks^32^, and later menopause^33–36^, as would be expected if puberty triggers a resumption of mutation accumulation, and consequently ageing, in both germline and somatic tissues.

The dynamics of accumulation of germline and somatic mutations from birth through adolescence during normal ageing has yet to be studied in depth. Relevant data for somatic mutations comes from the analysis of tumor genomes, since most of the somatic mutations detected in tumors appear during normal aging, before the tumor cell expands clonally^52,53^. The frequency of somatic mutations determined from paired tumor and normal tissue samples from the same individuals^52,53^, cancer incidence rates^54^, and intrinsic mortality rates^51^ are all plateaued at their lowest levels from approximately ages 5–14 years; they all then increase exponentially from late adolescence onward. Three of these four studies^51,53,54^ estimated the post-adolescence doubling time as approximately every eight years. The loss of health due to acquiring any chronic morbidity (i.e. the end of the healthspan) also begins in the teenage years and doubles approximately every eight years^55^. Thus DNA damage rates may be the major underlying determinant of all-cause mortality rates and chronic morbidity onset rates throughout the lifespan^54^.

As early development proceeds, declines in cell growth and proliferation rates should be accompanied by further declines in DNA damage rates. Once damage rates fall sufficiently, DNA repair systems may be sufficiently robust to keep up with the damage, resulting in mutation burdens and morbidity and mortality rates all being plateaued and at their lowest levels during the prepubertal years. After puberty, both metabolic rate, which correlates with DNA damage rates, and DNA repair genes’ expression levels decline with age^27,56^ and their rates and relative levels of decline are likely to vary between individuals, due to both heritable genetic factors and differences in environmental exposures, including diet, exercise, other lifestyle choices, and basic socioeconomic factors (income and wealth, education, and occupation). This range of influences, many modifiable by personal choice, likely produces substantial inter-individual variation in both germline and somatic mutation accumulation rates and, therefore, rates of ageing. However, significant variation in healthspan and lifespan may be expected even among individuals with identical puberty timing and mutation accumulation rates, if mutations are randomly distributed across the genome, only occasionally having pathogenic consequences.

While investigations of the causes of variation in the rate of ageing in adult populations are likely to lead to novel therapies to postpone frailty and extend the human healthspan, further study of the effects of puberty on mutation accumulation rates may also lead to important medical breakthroughs. Direct measurements of somatic mutation levels in normal (non-tumor) tissues from healthy subjects throughout childhood and adolescence are needed to either build support for or refute the hypothesis that somatic mutation levels are plateaued prepubertally. The well-documented plateaus in mortality rate found in several contexts, e.g. in humans during the prepubertal years^51^ and at age 105 or older^57^, and in hydra^58,59^ and asexual planaria^60^ throughout life, may share gene expression profiles that robustly maintain the integrity of the genome (or at least prevent its further deterioration) and maintain other aspects of homeostasis as well^4^, effectively putting ageing on hold.

Interventions in adults directed toward returning mutation accumulation rates to the negligible or very low levels that may be present prepubertally would be expected to have broad benefits, greatly lowering the risks for multiple ageing-related diseases and dramatically extending the human healthspan. Perhaps a relatively small number of genes that are master regulators of gene networks maintaining genome stability and homeostasis generally are downregulated at puberty, but can be reprogrammed^61^ or otherwise coaxed back to their prepubertal levels of activity by a combination of lifestyle, dietary, and/or pharmacological interventions.

## Methods

### Characteristics of the subjects

In the early 1980s, the 46 three-generation Utah CEPH families were contacted and enrolled in a project to build the first comprehensive human genetic linkage map^41,42^. Each Utah CEPH family consists of 4–16 siblings in the youngest generation (Generation III), their two parents (Generation II), and two to four grandparents (Generation I), as shown in Supplementary Fig. 1. Here we study cross-sectionally collected germline autosomal age-adjusted mutation rates (AAMRs) in 122 Generation I individuals (61 women and 61 men; demographic characteristics presented in Supplementary Table 1) from 41 of the families, in relation to those same Generation I individuals’ lifespans, cause-specific mortality, cancer incidence, and the women’s reproductive spans and age at menarche. We also analyze the timing and rate of accumulation of germline autosomal mutations in 40 Generation II parental couples, based on the detection of *de novo* mutations in the blood DNAs of their 350 Generation III offspring, by WGS analyses of all 430 of these Generations II and III subjects. No other traits of Generation II and III subjects were investigated in the current study. DNA was extracted from blood samples collected in the early 1980s and/or early 2000s.

In developing genetic linkage maps, the large sibship sizes of the Utah CEPH families allowed the segregation of genetic markers to be replicated in informative families, and the inclusion of grandparents helped in assigning alleles to maternal and paternal chromosomes (phasing)^41,42^. The families were not selected for any disease, but for large sibship sizes. Selecting for large sibships may select somewhat for higher than average fertility, and selecting for living grandparents may select somewhat for higher than average lifespan; however, large sibships are common in Utah, and more than half of the grandparents were younger than age 72 at the time of the initial enrollment. Therefore, these families are unlikely to be strongly enriched for factors contributing to longer reproductive lifespans and longer life. Furthermore, since the same selection criteria were applied across all collected families, these criteria should not introduce any biases for the current study. Analysis of DNA sequence polymorphisms^62^ available from the WGS data showed that all 122 Generation I Utah CEPH individuals included in this study are of European descent.

### Age-adjusted germline mutation rates

*De novo* mutations in the germ cells of parents can be found by WGS of DNA extracted from somatic tissue (e.g. blood samples) from parents and offspring, identifying high-confidence sequence changes in the offspring not present in either parent, and attributing each mutation to the parental germline in which it arose^40,63,64^. The number of germline mutations increases with parental age in both sexes, with higher absolute levels and higher rates of accumulation in males, and mutation counts varying more than two-fold between age-matched individuals of the same sex^40,63,64^.

WGS of blood DNA from 603 individuals from 41 three-generation families, identification of autosomal *de novo* mutations (single base substitutions and insertions and deletions of length 10 base pairs or less) in Generation II, and specific attribution of each mutation to the germline of a Generation I individual is described by Sasani and colleagues^40^; an example of this procedure is presented in Supplementary Fig. 1. Instead of simple counts of Generation I germline mutations, we analyzed germline mutation *rates*, obtained by dividing the number of mutations by the callable portion of the subject’s genome (number of autosomal mutations/number of diploid autosomal callable base pairs), to adjust for minor differences between subjects in the portion of the genome that met our requirements for validating mutations (Sasani *et al*.^40^, pp. 14–16). We then regressed germline autosomal mutation rates on parental age using a generalized linear model and used the resulting residuals to represent age-adjusted mutation rates (AAMRs).

### Outcomes

Generation I subjects were linked to the Utah Population Database (UPDB), a large and comprehensive resource of linked population-based information for demographic, genetic, and epidemiological studies (https://uofuhealth.utah.edu/huntsman/utah-population-database/acknowledging-updb.php). The UPDB is a dynamic genealogical and medical database that receives annual updates of Utah birth, death, and health records. Mortality was ascertained based on Utah death certificates linked to the UPDB. Causes of death were available in International Classification of Diseases (ICD) codes version 9–10 and aggregated into larger categories representing the leading causes of deaths. Cancer incidence records were drawn from the Utah Cancer Registry (https://uofuhealth.utah.edu/utah-cancer-registry/).

Fertility in Generation I women was assessed by parity (number of live births) and ALB, both derived from the UPDB. Since fertility in healthy women does not begin to decline with age until age 30^12–14^, differences between healthy women in normal rates of reproductive ageing will only be reflected by differences in the ALB when the ALBs in the studied cohort are ≥ age 30. Conversely, among healthy women whose ALB was <30, the variation in ALB attributable to inter-individual variation in normal rates of reproductive ageing is expected to be zero (i.e., variation in ALBs <30 is more likely to be secondary to personal choices and/or specific pathologic processes such as polycystic ovary syndrome or endometriosis, than to variation in ageing rates). Self-reported age at menarche (answer to the question, “At what age did your menstrual periods start [age in years]?”) was provided by the Utah Genetic Reference Project (UGRP).

### Statistical analysis

Full sample and sex-specific Cox proportional hazard models with adjustments for subject’s birth year were used to estimate the effects of AAMRs on mortality and cancer risks, expressed as hazard rate ratios (HR) in Generation I individuals. Results for both sexes combined were adjusted for sex. Time was measured in years from the Generation I individual’s parental age at the birth of the index child to time of death (n = 120) or last known living dates up to 2018 (n = 2). Cause-specific mortality was analyzed by fitting cause-specific hazard regression models with Cox regression, treating failures from the cause of death of interest as events and failure from other causes of deaths or those still living as right-censored^65^. For cancer incidence analyses, Cox proportional hazard models were used to assess the effect of AAMRs as a continuous or categorical variable on cancer hazard rate ratios (HR) of the Generation I subjects, additionally adjusted for birth year and parental age.

Fertility analyses were restricted to the 53 women with an ALB ≥ 30 years, since cessation of childbearing prior to age 30 is unlikely due to reproductive ageing^12–14^. Poisson regression models were used to assess the effect of AAMRs on the number of live births to Generation I women. Logistic regression models were used to assess the effect of AAMRs on ALB where ALB was treated as a categorical variable (i.e. <25^th^ percentile). All fertility analyses in these Generation I women were adjusted for subject’s birth year.

The association of AAMRs with age at menarche in the 20 Generation I women for whom menarcheal age was available was tested by a Pearson correlation (r) analysis, using 5000 bootstrapped samples, given the small sample size. The significance of r is assessed using an empirical (bootstrapped) p value and confidence intervals. The difference in mean age at menarche between women with AAMRs ≥ 50^th^ percentile vs. women below the 50^th^ percentile was also analyzed.

Statistical adjustments for multiple comparisons were not included in our analyses, because these planned comparisons^66^ all focused on a single prediction - that higher AAMRs would be associated with the earlier occurrence of key milestones of aging (i.e. cessation of childbearing, death, and cancer incidence). All comparisons showing unadjusted significant differences supported the prediction, strongly suggesting that the low unadjusted p-values we observed in several comparisons are unlikely due to chance alone.

### Ethical approvals

All studies were approved by the Institutional Review Board of the University of Utah (IRB_00021454 and IRB_00011975), and by the Resource for Genetic and Epidemiologic Research (RGE), University of Utah. All methods were performed in accordance with relevant guidelines and regulations. All participants provided signed, paper-based informed consent.

## Supplementary information

Supplementary information.

Supplementary information5.

## Data Availability

Whole genome sequencing data. Aligned sequencing reads (in CRAM format) and variant calls (in VCF format) are available under controlled access at the SRA and dbGaP, with accession phs001872.v1.p1. Files describing *de novo* mutations (DNMs) observed in Generation II individuals (offspring) and attributed to the germlines of Generation I individuals (parents) have been deposited on GitHub at https://github.com/quinlan-lab/ceph-dnm-manuscript ^40^. Life history and clinical outcomes data. Utah Population Database (UPDB) data contributed to this project using birth and death records and family data that include Protected Health Information and individual identifiers. Special attention is given to protect individuals and their information contained within the UPDB and the organizations that contribute data while also allowing access to researchers. Accordingly, the Utah Resource for Genetic and Epidemiologic Research (RGE), established in 1982 by Executive Order of the Governor of Utah, administers access to the UPDB through a review process of all proposals using UPDB data. The protection of privacy and confidentiality of individuals represented in these records has been negotiated with agreements between RGE and data contributors. Data from the UPDB is available only for approved health-related research studies and access is project-specific and granted after review and approval by an RGE oversight committee and the University of Utah’s IRB. This process allows researchers with approved protocols to use the data, a process that has proven effective and successful as evidenced by hundreds of approved studies that have relied on the UPDB. Editors and reviewers of our manuscript who feel that they need access to UPDB source data beyond that which is contained in the manuscript, in order to evaluate our study, are requested to contact the corresponding author. Once those needing access sign an RGE Confidentiality Agreement and the request for data access is promptly reviewed and approved, access to the relevant data will be granted. Code for mortality, cancer incidence, and fertility analyses. This is provided at the end of the Supplementary Information.
