# Supplementary material for: Germline mutation rates in young adults predict longevity and reproductive lifespan
Source: Sci Rep. 2020 Jun 19;10:10001. doi: 10.1038/s41598-020-66867-0 (PMC7305191; doi:10.1038/s41598-020-66867-0)
Supplement: Supplementary file 2 — Supplementary information5. [file 41598_2020_66867_MOESM2_ESM.docx]

# **Supplementary Information**

1. **Supplementary Tables**

**Supplementary Table 1: Demographic Characteristics of Utah CEPH Grandparents (n=122)**

**Supplementary Table 2: Median age of subjects in each category of age-adjusted mutation rates**

**Supplementary Table 3: Associations of [germline mutation rate / parental age] with mortality in 122 Generation I individuals**

**Supplementary Table 4: Associations of germline mutation rates with cancer risk**

1. **Supplementary Figures**

**Supplementary Figure 1: Pedigree structure of Utah CEPH three-generation families, and strategy for identifying germline mutations in Generation I individuals**

**Supplementary Figure 2: Generation II parental couples with similar germline autosomal mutation accumulation rates vary ~5 years in the paternal age at which equivalent numbers of mutations are accumulated**

**Supplementary Figure 3: Model for germline mutation accumulation with age, with likely sources for inter-individual variation in age-specific mutation levels**

1. **Code for CEPH mortality, cancer incidence, and fertility analyses**

**Supplementary Tables**

|  | Men (N = 61) | Women (N = 61) |
| --- | --- | --- |
| Number of germline autosomal mutations | 51.1 ± 11.9 | 12.9 ± 4.3 |
| Germline mutation rate* | (9.9 ± 2.3) x 10^-9^ | (2.5 ± 0.8) x 10^-9^ |
| Birth year | 1909.7 ± 7.7 | 1912.6 ± 7.2 |
| Born in Utah |  |  |
| - Yes | 39 (63.9%) | 36 (59.0%) |
| - No | 18 (29.5%) | 24 (39.3%) |
| - Unknown | 4 (6.6%) | 1 (1.6%) |
| European descent (by genotype analysis, ref. 62) |  |  |
| - Yes | 61 (100%) | 61 (100%) |
| Hispanic |  |  |
| - No | 48 (78.7%) | 50 (82.0%) |
| - Yes | 1 (1.6%) | 2 (3.3%) |
| - Unknown | 12 (19.7%) | 9 (14.8%) |
| Age at index births (Parental Age) | 29.1 ± 5.6 | 26.3 ± 5.2 |
| Women with age at last birth ≥ 30 years (n = 53) |  |  |
| - number of live births | - | 6.3 ± 2.5 |
| - age at last birth | - | 38.7 ± 4.6 |
| - live births with no missing birth dates | - | 46 (86.8%) |
| - live births with ≥ one missing birth date | - | 7 (13.2%) |
| Age at menarche (n = 20) | - | 12.8 ± 0.9 |
| Cancer diagnosis |  |  |
| - No | 43 (70.5%) | 45 (73.8%) |
| - Yes | 18 (29.5%) | 16 (26.2%) |
| Age at first cancer diagnosis | 76.4 ± 8.9 | 75.6 ± 11.3 |
| Death year | 1995.8 ± 8.0 | 2000.6 ± 9.7 |
| Died in Utah |  |  |
| - Yes | 46 (75.4%) | 48 (78.7%) |
| - No | 5 (8.2%) | 9 (14.8%) |
| - Unknown | 10 (16.4%) | 4 (6.6%) |
| Censor in 2018 |  |  |
| - Alive | 1 (1.6%) | 1 (1.6%) |
| - Death | 60 (98.4%) | 60 (98.4%) |
| - Infection | 0 (0.0%) | 1 (1.6%) |
| - Cancer | 4 (6.6%) | 4 (6.6%) |
| - Endocrine/Metabolic | 2 (3.3%) | 4 (6.6%) |
| - Mental/Psychoneurotic | 1 (1.6%) | 2 (3.3%) |
| - Nervous system | 1 (1.6%) | 2 (3.3%) |
| - Circulatory system | 30 (49.2%) | 24 (39.3%) |
| - Respiratory | 4 (6.6%) | 2 (3.3%) |
| - Digestive System | 1 (1.6%) | 2 (3.3%) |
| - Other | 3 (4.9%) | 6 (9.8%) |
| - Unknown | 14 (23.0%) | 13 (21.3%) |
| *(#germline autosomal mutations / #diploid autosomal callable base pairs) | | |
| **Supplementary Table 1** Demographic Characteristics of Utah CEPH Grandparents (n = 122) | | |

|  | **Quartiles** | | | |  | **Tertiles** | | |
| --- | --- | --- | --- | --- | --- | --- | --- | --- |
|  | < 25^th^ percentile | 25^th^-50^th^ percentile | >50^th^-75^th^ percentile | > 75^th^ percentile |  | < 33^rd^ percentile | 33^rd^ – 66^th^ percentile | > 66^th^ percentile |
| **Males** | 27.5 | 30 | 27.7 | 28.3 |  | 27.4 | 29 | 27.7 |
| **Females** | 25.9 | 25.6 | 24.5 | 26.7 |  | 25.9 | 25.1 | 26.6 |
| **Supplementary Table 2** Median age of subjects in each category of age-adjusted mutation rates | | | | | | | | |

| **Age-adjusted germline mutation rates** |  | **Both sexes** | |  | **Males** | |  | **Females** | |
| --- | --- | --- | --- | --- | --- | --- | --- | --- | --- |
|  |  | **HR (95% CI)** | **p** |  | **HR (95% CI)** | **p** |  | **HR (95% CI)** | **p** |
| Continuous |  | 1.97 (1.12, 3.46) | ***0.019*** |  | 1.54 (1.05, 2.25) | ***0.025*** |  | 1.26 (0.92, 1.72) | 0.158 |
| 25^th^-50^th^ percentile |  | 1.28 (0.75, 2.19) | 0.359 |  | 0.91 (0.43, 1.95) | 0.816 |  | 2.10 (0.94, 4.67) | 0.069 |
| >50^th^-75^th^ percentile |  | 1.61 (0.95, 2.73) | 0.080 |  | 1.74 (0.83, 3.65) | 0.140 |  | 1.42 (0.64, 3.15) | 0.392 |
| > 75^th^ percentile |  | 1.73 (1.02, 2.93) | ***0.042*** |  | 1.84 (0.84, 4.05) | 0.130 |  | 1.81 (0.85, 3.89) | 0.125 |
| Trend test |  | 1.20 (1.02, 1.42) | ***0.028*** |  | 1.29 (1.00, 1.66) | ***0.048*** |  | 1.15 (0.91, 1.45) | 0.235 |
| Hazard ratios (HR) and 95% Confidence Intervals (CI) were estimated using Cox proportional hazard models to assess the association of germline mutation rates per parental age [(#autosomal mutations / diploid autosomal callable base pairs) / (Parental Age)] as a continuous variable and as quartiles with all-cause mortality risk of CEPH Generation I subjects and additionally adjusted for birth year. Time was measured in years from Parental Age to time of death or last known living dates (as of 2018). Analyses were performed for both sexes combined and for each sex separately. The first row (Continuous) presents the impact on the Hazard Ratio (HR) of a one standard deviation increase in age-normalized mutation rates. The second, third, and fourth rows present the mortality risks to subjects with increasing quartiles of age-normalized mutation rates, expressed relative to the mortality risks for the lowest quartile (<25^th^ percentile). | | | | | | | | | |
| **Supplementary Table 3** Associations of [germline mutation rate / parental age] with mortality in 122 Generation I individuals | | | | | | | | | |

| **Age-adjusted germline mutation rate** | **Both sexes** | | | **Males** | | | **Females** | | |
| --- | --- | --- | --- | --- | --- | --- | --- | --- | --- |
|  |  |  |  |  |  |  |  |  |  |
|  | **Number of cases / number at risk** | **HR (95% CI)** | **p** | **Number of cases / number at risk** | **HR (95% CI)** | **p** | **Number of cases / number at risk** | **HR (95% CI)** | **p** |
| **Continuous*** | **34/122** | **1.19 (0.83, 1.69)** | **0.340** | **18/61** | **1.47 (0.87, 2.48)** | **0.149** | **16/61** | **0.74 (0.44, 1.24)** | **0.259** |
| **< 33^rd^ percentile** | **10/40** | **1** | **-** | **5/20** | **1** | **-** | **5/20** | **1** | **-** |
| **33^rd^ – 66^th^ percentile** | **10/40** | **1.03 (0.42, 2.50)** | **0.952** | **3/20** | **0.70 (0.16, 2.97)** | **0.626** | **7/20** | **1.08 (0.31, 3.74)** | **0.897** |
| **> 66^th^ percentile** | **14/42** | **1.50 (0.66, 3.41)** | **0.334** | **10/21** | **2.53 (0.85, 7.50)** | **0.094** | **4/21** | **0.60 (0.15, 2.37)** | **0.463** |
| **Trend test** | **-** | **1.24 (0.81, 1.88)** | **0.318** | **-** | **1.73 (0.96, 3.12)** | **0.071** | **-** | **0.77 (0.40, 1.48)** | **0.440** |
| Cox proportional hazard models were used to assess the effect of AAMRs as a continuous or categorical variable on cancer hazard rate ratios (HR) of CEPH Generation I subjects, and additionally adjusted for birth year and parental age. The cancer hazard rate for a given group is estimated by first measuring the number of incident cancer cases occurring in a unit of time among those who have not yet been diagnosed; then the hazard rates of two groups are compared to generate the HR. Time was measured in years from parental age to time of death or last known living dates (up to 2018) or first cancer diagnosis, whichever occurred first. The first row (Continuous) presents the effects on cancer HR of a one standard deviation increase in AAMRs. The third and fourth rows present the cancer HR for subjects in the middle and top tertiles for AAMRs relative to subjects in the bottom tertile. (Tertiles rather than quartiles were analyzed to provide more stable risk estimates, given the small number of cancer cases.) The cut points for AAMRs are: Males: 33% = 0.9104878, 66% = 0.7600077; Females: 33% = 0.34799546, 66% = 0.30450212. HR (95% CI): Hazard Ratio and 95% Confidence Interval. | | | | | | | | | |
| **Supplementary Table 4** Associations of age-adjusted germline mutation rates with cancer Hazard Ratios | | | | | | | | | |

**
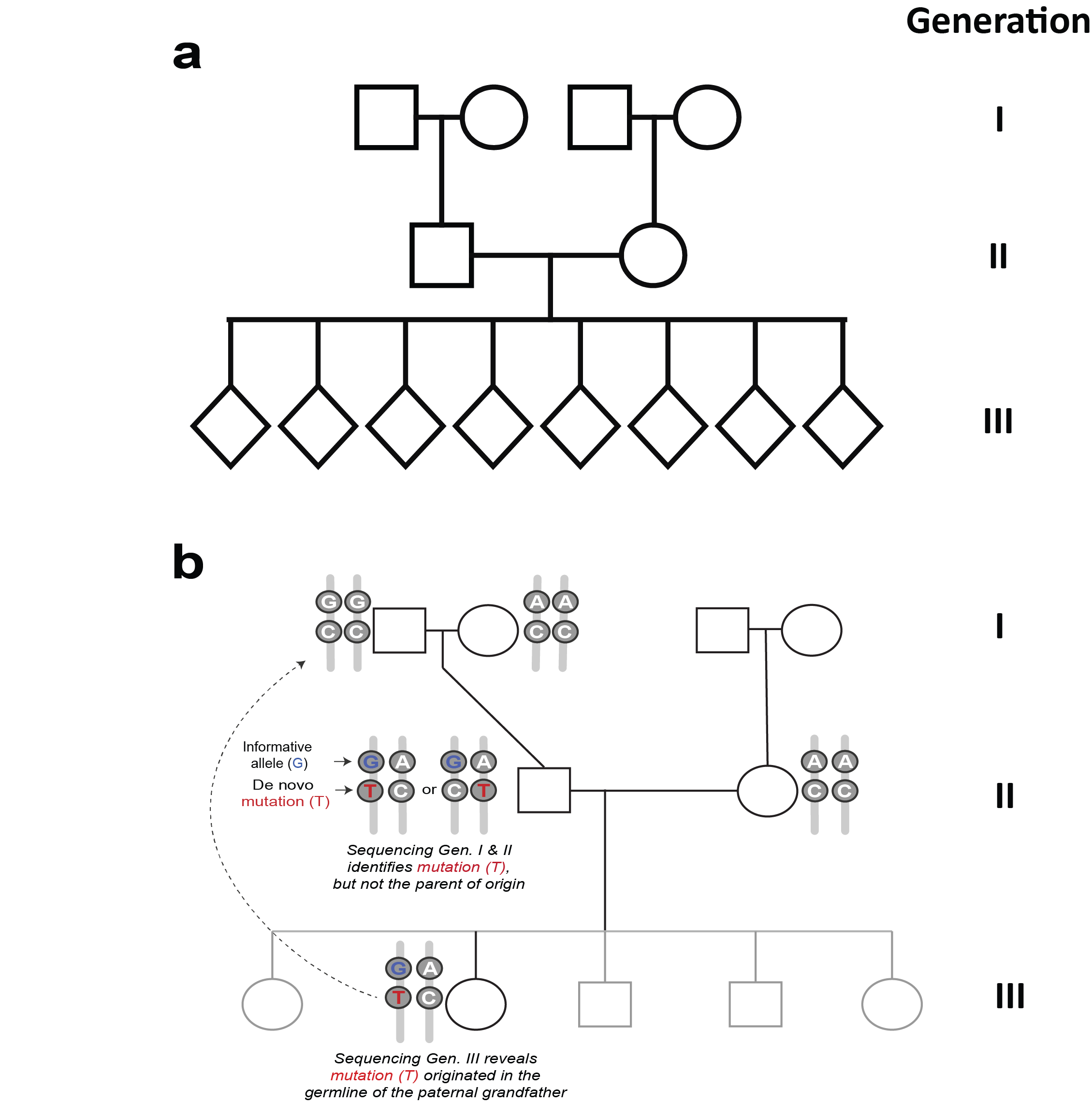
Supplementary Figures**

**Supplementary Figure 1 a.** Pedigree structure of Utah CEPH three-generation families, Circles denote females, squares males, and diamonds third-generation grandchildren of either sex. Generation III sibship size ranged from 4-16. **b.** An example of the procedure used to identify mutations that originated in the germlines of Generation I individuals. Whole genome sequencing (WGS) of blood DNAs from Generations I and II identifies *de novo* mutation (T) in the Gen. II man, which could have originated in either of his parents’ germlines. However, in analyzing WGS from Gen. 3, since we know the Gen. II woman transmitted the AC haplotype, we can infer that mutation (T) changed a GC haplotype to a GT haplotype in a gamete of the paternal grandfather, and therefore count that mutation towards the grandfather’s observed germline mutation rate. Inspection of additional informative genetic markers along the chromosome, upstream and downstream of the DNM, provided further confirmation of the parental haplotypes, allowing us to exclude a recombination event in the germline of the Gen. II man as the origin of the GT haplotype found in the Gen. III daughter [40]. Additional criteria were applied to exclude from consideration early post-zygotic mutations in Generation II individuals (Sasani et al. [40], pp. 9-10). For the current study, analyses of lifespan, cause-specific mortality, and cancer incidence in both sexes, and reproductive spans and age at menarche in women, were limited to Generation I individuals.

**
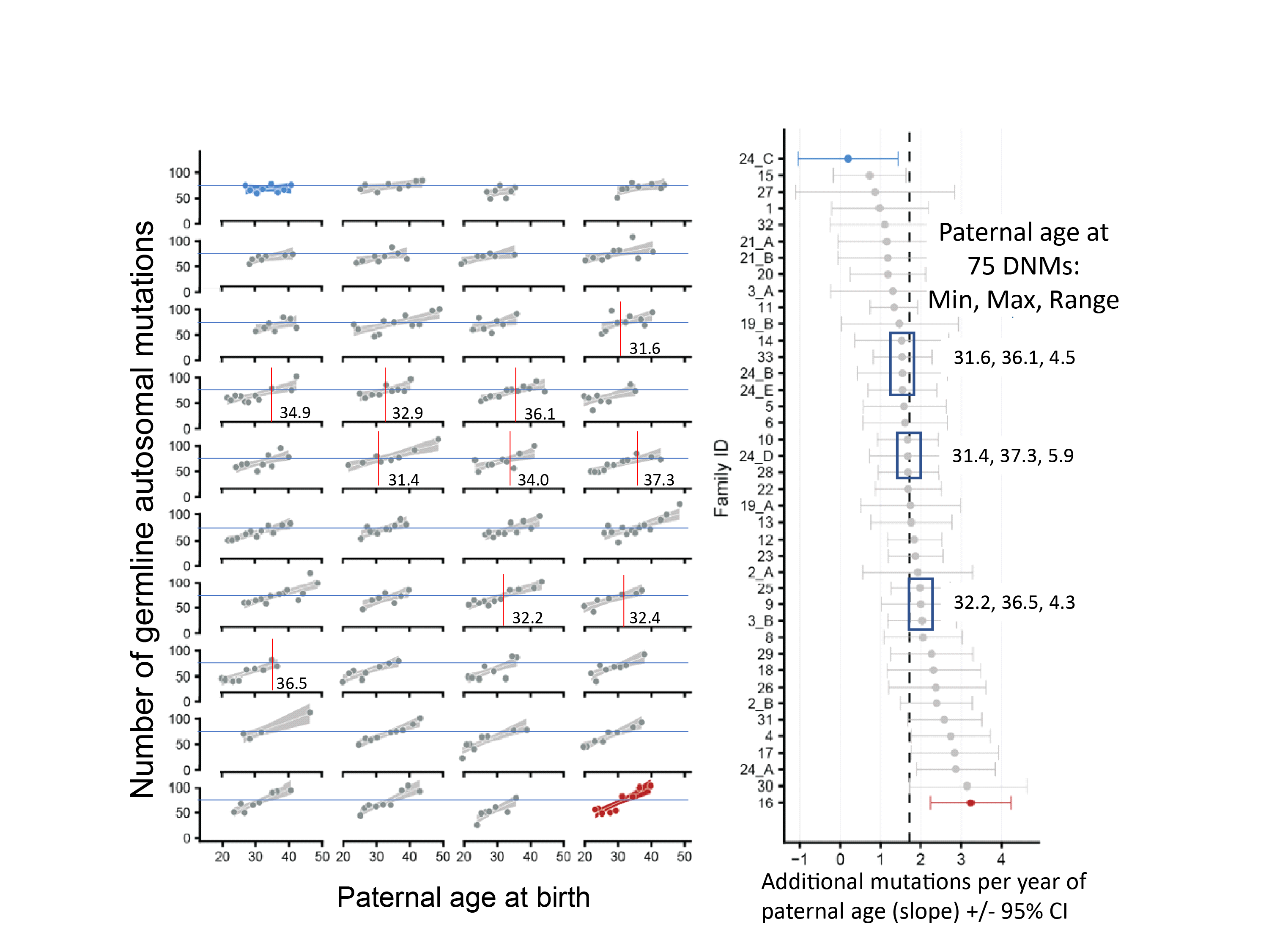
**

**Supplementary Figure 2** Generation II parental couples with similar germline autosomal mutation accumulation rates vary ~5 years in the paternal age at which equivalent numbers of mutations are accumulated. Adapted from reference 40, Fig. 3c and 3d. Left panel: longitudinal data for *de novo* mutations (DNMs) discovered in 40 Generation III sibships, attributable to the germlines of the corresponding 40 Generation II parental couples. Each of the 40 plots shows the numbers of DNMs found in each sibling in a single sibship. The plots are presented, left to right and top to bottom, in order of increasing germline mutation accumulation rates. The mutation counts mainly reflect the fathers’ mutations, as the fathers have higher baseline levels and higher mutation accumulation rates than the mothers. In each row the horizontal blue line corresponds to 75 mutations accumulated, and the vertical red lines identify the paternal age at which the 75 mutations are projected to have accumulated. These paternal ages, derived from equations fitting linear regression lines to each sibship’s data points, are written to the right of each vertical red line. Right panel: the mutation accumulation rates for the 40 couples are listed from top to bottom in order of increasing rate. Note the more than 3-fold range of mutation accumulation rates. Blue boxes identify subsets of parental couples with very similar within-subset rates. To the right of each blue box, the minimum, maximum, and range for the paternal age needed to accumulate 75 mutations is given. The average for this range, across the three subsets, is 4.9 years, suggesting that the age at which adult germline mutation accumulation rates become established varies approximately five years, which is also the reported range for the age of onset of puberty [50].


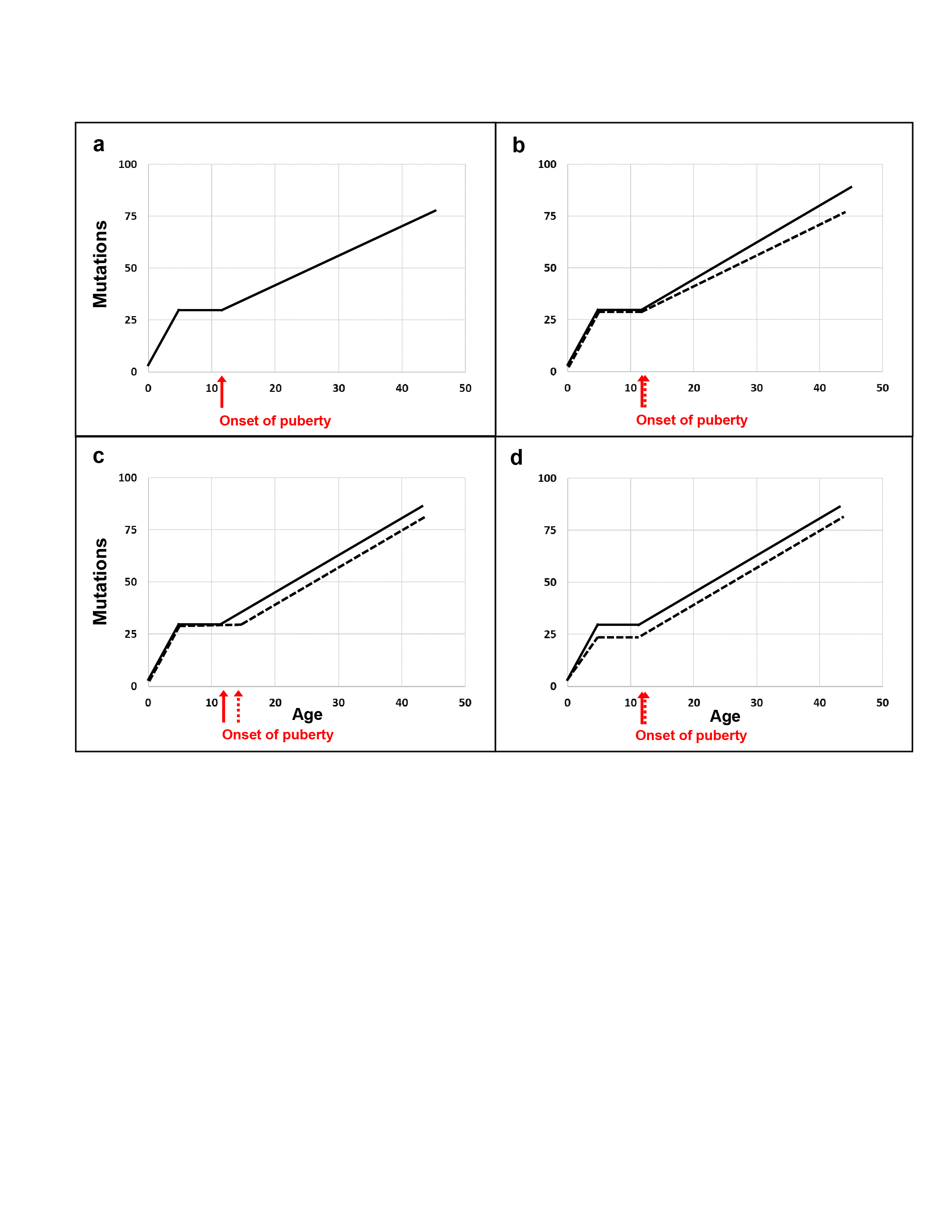


**Supplementary Figure 3** Model: expected longitudinal data for germline mutation burdens within individuals, including likely sources for variation in age-specific mutation counts. **(a)** High mutation accumulation rates in early development [48], followed by a prepubertal plateau and a puberty-induced resumption of mutation accumulation [49]. **(b)** Two individuals with the same age of onset of puberty but different postpubertal mutation accumulation rates. **(c)** Two individuals differing in age of onset of puberty but with the same postpubertal mutation accumulation rate. **(d)** Two individuals differing in the level of the prepubertal plateau. (Male germline mutation rates are modeled here; the same principles apply to the modeling of female germline mutation rates.)
